# Re-description of the type species of the genera *Ganesella* Blanford, 1863 and *Globotrochus* Haas, 1935; with description of a new *Ganesella* species from Thailand (Eupulmonata, Camaenidae)

**DOI:** 10.3897/zookeys.870.36970

**Published:** 2019-08-07

**Authors:** Chirasak Sutcharit, Thierry Backeljau, Somsak Panha

**Affiliations:** 1 Animal Systematic Research Unit, Department of Biology, Faculty of Science, Chulalongkorn University, Bangkok 10330, Thailand Chulalongkorn University Bangkok Thailand; 2 University of Antwerp, Evolutionary Ecology Group, Groenenborgerlaan 171, B-2020 Antwerp, Belgium University of Antwerp Antwerp Belgium; 3 Royal Belgian Institute of Natural Sciences (JEMU & BopCo), Vautierstraat 29, B-1000 Brussels, Belgium Royal Belgian Institute of Natural Sciences Brussels Belgium

**Keywords:** anatomy, Cerastidae, Indochina, Orthurethran, Southeast Asia, synonym, systematics, tree snail

## Abstract

The taxonomy of the speciose genus *Ganesella* W.T. Blanford, 1863 and the endemic genus *Globotrochus* Haas, 1935 is unclear since the anatomical characters of the the type species of these two genera have never been reported before. Therefore, the present paper provides the first anatomical descriptions of the reproductive apparatus, pallial system and radula of *Helix capitium* Benson, 1848 and *Helix onestera* Mabille, 1887, the respective type species of *Ganesella* and *Globotrochus*. In addition, *Ganesella rhombostoma* (Pfeiffer, 1861) and *Ganesella carinella* (Möllendorff, 1902) from Thailand are re-described, and a new species, *Ganesella halabalah* Sutcharit & Panha, **sp. nov.**, from southern Thailand is described. This new species differs from all others by having a larger shell, an obtuse apex and an aperture lip with a prominent beak-like deflection.

## Introduction

The Camaenidae is one of the most speciose pulmonate snail families in Asia, and shows an astonishing diversity of taxa with different shell shapes, sizes and ecological characteristics (e.g., Pilsbry 1895, Solem 1959, Richardson 1985). Hitherto, camaenid taxonomy and classification has relied heavily on the shell morphology and, to a far lesser extent, on the reproductive anatomy. However, pulmonate shell characters are poor taxonomic markers as they often show convergent evolution and/or plastic features. The Camaenidae of Southeast Asia, including eastern India, Japan, Taiwan and southern China, are ground-dwelling or arboreal snails with relatively small to medium-sized, dextral or sinistral, trochoid shells. Traditionally, they were all assigned to the genus *Ganesella* W.T. Blanford, 1863.

The (sub)generic name *Ganesella* was first coined without any description or definition to accommodate *Helix capitium* Benson, 1848 and *Helix hariola* Benson, 1856 (Blanford 1863). As such, the definition of this (sub)genus has remained unclear (Zhou et al. 2011), the more so as the genital anatomy of *Helix capitium* was unknown until now (Schileyko 2003). Hence, the name *Ganesella* has been applied to a heterogeneous assemblage of what appears now to be at least four nominal subgenera (Thiele 1931, Zilch 1960, Richardson 1985). Unfortunately, there is little, if any, anatomical information on these (sub)genera.

The first data on the genital apparatus of *Ganesella* were published by Pilsbry (1895) for *Helix japonica* Pfeiffer, 1847, the type species of the genus *Satsuma* Adams, 1868. This genus was considered for a long time as a junior synonym of *Ganesella* (Pilsbry 1895, Thiele 1931, Zilch 1960). Yet, subsequent anatomical and molecular evidences have confirmed that *Satsuma* is to be treated as a distinct genus (Azuma 1995, Schileyko 2003, Wu et al. 2008), and is comprised of the species from Japan, Taiwan and southern China that were formerly assigned to *Ganesella*. As a consequence, the current interpretation of *Ganesella* confines this genus geographically to east of India and Southeast Asia. However, a general and consistent delimitation of *Ganesella* in terms of its genital features is still lacking, not in the least because the genital anatomy of its type species, *Helix capitium* was unknown until now (see below). Hence, species were assigned to *Ganesella* on the basis of shell characters only, and this has led to several misclassifications. A case in point is *Ganesella brevibarbis* (Pfeiffer, 1859) from China, which, after anatomical study, appeared to belong to the genus *Plectotropis* Martens, 1860 in the family Bradybaenidae (Albers 1860, Zhou et al. 2011).

The recent revision of the Camaenidae by Schileyko (2003) has raised several subgenera of *Ganesella* to full genus rank (*Liocystis* Mörch, 1872, *Coliolus* Tapparone-Canefri, 1887, *Coniglobus* Pilsbry & Hirase, 1906 and *Globotrochus* Haas, 1935), but this was still based only on shell characters. Hence, the first step towards a sound taxonomic revision of *Ganesella* in Indochina is to provide comparative anatomical data of the type species of the different genus-level taxa involved. The present study does so for the nominal genera *Ganesella* and *Globotrochus*, with their respective type species *Helix capitium* and *Helix onestera* Mabille, 1887. In addition, *Ganesella rhombostoma* (Pfeiffer, 1861) and *Ganesella carinella* (Möllendorff, 1902) from Thailand are re-described, and a new species from southern Thailand is described.

## Materials and methods

Shells and living specimens were collected from various localities in Thailand and Vietnam. Live specimens were drowned in water and then fixed in 70% (v/v) ethanol for anatomical examination. Specimens were primarily identified using the publications of Benson (1848, 1856), Pfeiffer (1853), Pilsbry (1891, 1895) and Möllendorff (1898), and were also compared with the relevant type material in museum collections (see below). To study anatomy, three to ten specimens were dissected under a stereomicroscope. Drawings were made with a camera lucida. Adult shells were used to measure the shell height (**h**) and shell width (**w**), and to count the number of whorls. Radulae were examined under scanning electron microscopy (SEM; JEOL, JSM-5410 LV).

### Anatomical conventions and abbreviations

In the descriptions of the genitalia, the term ‘proximal’ refers to the region closest to the genital orifice, while ‘distal’ refers to the region furthest away from the genital orifice. The following abbreviations were used as defined by Pilsbry (1891, 1895) and Solem (1993): **a**, anus; **ag**, albumen gland; **at**, atrium; **au**, auricle; **e**, epiphallus; **fl**, flagellum; **fo**, free oviduct; **gd**, gametolytic duct; **gs**, gametolytic sac; **hd**, hermaphroditic duct; **hg**, hermaphroditic gland; **i**, intestine; **k**, kidney; **l**, lung; **mc**, mantle collar; **ov**, oviduct; **p**, penis; **pn**, pneumostome; **pp**, penial pilaster; **pr**, penial retractor muscle; **puv**, pulmonary vein; **pv**, penial verge; **r**, rectum; **ur**, ureter; **v**, vagina; **vd**, vas deferens; **ve**, ventricle; **vp**, vaginal pilaster.

### Institutional abbreviations

**CUMZ**, Chulalongkorn University, Museum of Zoology, Bangkok; **FMNH**, Field Museum of Natural History, Chicago; **MNHN**, Muséum National ďHistoire Naturelle, Paris; **NHMUK**, The Natural History Museum, London; **NHMW**, Naturhistorisches Museum, Wien; **RBINS**, Royal Belgian Institute of Natural Sciences, Brussels; **SMF**, Forschungsinstitut und Naturmuseum Senckenberg, Frankfurt am Main; **UMZC**, University Museum of Zoology Cambridge, Cambridge; **ZMB**, Museum für Naturkunde, Humboldt University, Berlin.

## Systematics

### Family Camaenidae Pilsbry, 1895

#### Genus Ganesella

Taxon classificationAnimaliaStylommatophoraCamaenidae

Blanford, 1863

CB73D722D6955ABD9CAEDC6C69062B3A

Helix (Ganesella) W.T. Blanford, 1863: 86.
Trochomorphoides Nevill, 1878: 80. Type species: Helix acris Benson, 1859, by original designation.
Darwininitium Budha & Mordan in Budha et al. 2012: 21. Type species: Darwininitium shiwalikianum Budha & Mordan in Budha et al. 2012, by original designation. New synonym.

##### Type species.

*Helix capitium* Benson, 1848 by subsequent designation (Pilsbry 1895: 168).

##### Description.

Shell more or less trochoid, moderately thin to solid, with 4–6 convex whorls. Last whorl rounded to angular, with or without a peripheral keel, a little descending in front. Colour light monochrome or with a few dark bands and/or spots and streaks. Embryonic shell smooth. Post apical whorls with irregular, thin, radial ridges and spiral lines (smooth below peripheral angle or keel). Aperture widely ovate, moderately oblique with variously reflected margins. Umbilicus narrow, but open, rarely closed. Shell height ranged from 4.5 to 25.0 mm and shell width ranged from 5.5 to 27.0 mm.

Genitalia typical of camaenids, without accessory organs on penis or vagina. Penis short to long, with small to large penial verge, but no penial appendix; epiphallus and flagellum short.

Radular teeth triangular to spatulate, central tooth unicuspid, lateral and marginal teeth tricuspid.

##### Remarks.

The genus *Ganesella* s.s. differs from *Satsuma* s.s by having a penial verge, but no penial appendix. In contrast, *Satsuma* has a short to long penial appendix on the distal part of the penis, but lacks a penial verge. In addition, *Satsuma* occurs from Japan to Taiwan and southern China, while *Ganesella* occurs from South to Southeast Asia, including Japan (Azuma 1995, Schileyko 2003, Wu et al. 2008).

*Darwininitium shiwalikianum* Budha & Mordan, 2012, the type species of the monotypic genus *Darwininitium* Budha & Mordan, 2012, appears conchologically identical to *Helix capitium*, the type species of *Ganesella* (see also Budha et al. 2016). Therefore, the genus *Darwininitium*, which was originally described from central Nepal, is here tentatively regarded as a junior subjective synonym of the camaenid genus *Ganesella*. If this is confirmed, then *Darwininitium* was erroneously assigned to the family Cerastidae and as such, its pallial system with a long kidney and s-shape ureter would point to a sigmurethrous condition and not to a case of pseudosigmurethry within the Orthurethra (sensu Solem 1959 and see also Budha et al. 2016). These taxonomic conclusions appear to be supported by DNA sequence data (Budha et al. 2016).

#### Ganesella capitium

Taxon classificationAnimaliaStylommatophoraCamaenidae

(Benson, 1848)

43794688E83058DE807AEAACF0B8162B

Figs 2A, B , 8

Helix capitium Benson, 1848: 160. Hanley and Theobald 1870: 7, pl. 14, fig. 5. Tryon 1887: 74, pl. 14, fig. 99.
Helix (Planispira) capitium : Nevill 1878: 78.
Trochomorpha capitium : Morlet 1889: 124, 125.
Ganesella capitium : Pilsbry 1895: 170, pl. 55, fig. 18. Blanford 1903: 278. Gude 1914: 196, 197. Zilch 1960: 610, fig. 2140. Zilch 1966: 202. Richardson 1985: 132, 133.
Darwininitium shiwalikianum Budha & Mordan in Budha et al. 2012: 21–23, figs 2–4. Type locality: Kasara near Tamor Lake, Chitwan National Park, Central Nepal. New synonym.

##### Type locality.

Sicrigali province Bahar Indiae Orientalis [Bihar State, India].

##### Material examined.

**Type specimens.** Three syntypes of *Helix capitium* are in Benson’s collection. The specimen that closely matched with the measurement in the original description is designated here as the lectotype UMZC I.102385/1 (Fig. 2A, height 13.3 mm, width 13.5 mm), and the other two as paralectotypes UMZC I.102385/2–3 (2 shells; Fig. 2B, height 14.4 mm, width 13.8 mm).

##### Additional material.

Bahar Province, India: ZMB ex. Albers coll. 1 lot (1 shell). South India: NHMUK ex. Godwin-Austen coll. no. 501 (2 shells).

##### Remarks.

Budha et al. (2012) described *Darwininitium shiwalikianum* from Chitwan National Park, Nepal. The holotype has a trochoid, brownish shell with irregular opaque white spots. As such, it appears to be identical to the type specimens of *Ganesella capitium*. In addition, the type localities of *D. shiwalikianum* and *G. capitium* are geographically quite close to each other. Hence, in line with our earlier conclusions about the genus *Darwininitium*, we tentatively regard *D. shiwalikianum* as a junior subjective synonym of *G. capitium*.

Shell and genitalia have been described in detail by Budha et al. (2012). The unique and distinctive characters of *G. capitium* are its small, relatively elevated, trochoid shell. Aperture open sublaterally. Whorls slightly convex with wide and shallow suture. Last whorl angular with weak peripheral keel. Shell colour brownish with whitish-opaque, irregular, spots or streaks. Genitalia with short atrium; cylindrical, short penis, about half as long as the vagina, and proximally with blackish, spongy tissue. Epiphallus and flagellum together short, about as long as the penis, but flagellum longer than epiphallus. Internal wall of penis with numerous longitudinal pilasters.

#### Ganesella hariola

Taxon classificationAnimaliaStylommatophoraCamaenidae

(Benson, 1856)

800FAD1F3A185F7DB08EB97BDF901C94

Figs 2C–E , 8

Helix hariola Benson, 1856: 251. Pfeiffer 1860: 123, pl. 36, figs 21, 22. Hanley and Theobald 1870: 7, pl. 14, fig. 6.
Helix capitium var. hariola : Tryon 1887: 74, pl. 14, fig. 100.
Helix (Ganesella) hariola var. carinata Godwin-Austen, 1888: 242. Type locality: Khagan on Irrawaddy, and Hlindet, 1200 feet.
Ganesella capitium var. hariola : Pilsbry 1895: 170. Gude 1914: 197.

##### Type locality.

Thyet-Myo, prope ripas Irawadi fluvii [near the banks of the River Irrawaddy in Thayetmyo, Magway Region, Myanmar].

##### Material examined.

**Type specimens.** To stabilize the name, the syntype from Benson’s collection that most closely matched with the features and measurements of the original description is here designated as the lectotype UMZC I.104370/1 (Fig. 2C, height 11.6 mm, width 14.4 mm) of *Helix hariola* Benson, 1856. The other shells from the same lot hence become the paralectotypes UMZC I.104370/2–4 (3 shells; Fig. 2D, height 13.8 mm, width 16.2 mm).

##### Additional material.

**MYANMAR**: Thungadan, North Ava, Burma: syntype of Helix hariola var. carinataNHMUK 1906.2.2.176 (4 shells). Burma: NHMUK 1906.2.2.276. Pegu: NHMUK Salisbury coll. ex. Beddome (1 shell). North Chin Hills, Upper Burma: NHMUK 1893.12.6.30–4 (5 shells). Thyet-myo, Pegu, Burma: NHMUK 1906.2.2.109 (4 shells). Pegu, Burma: ZMB Paetel coll. (2 shells), Dunker coll. (1 shell). Pyintha, Mandalay, Burma: ZMB Notling coll. (3 shells). Popa View Point Resort, about 50 km southeast of Bagan (20°55'19.1"N, 95°12'41.9"E), Kyaukpandaung Township, Nyaung-U District, Mandalay Region: CUMZ 5134 (1 shell; Fig. 2E).

##### Description.

Shell small, dextral, thin and with a depressed trochoid. Apex acute; embryonic shell smooth with brownish colour. Whorls 5-6, increasing regularly, convex; suture wide and deep. Shell surface smooth or with fine growth lines. Last whorl large, very weekly angular to rounded; beneath convex; last whorl with brownish peripheral band. Shell brownish and translucent, with whitish-opaque, irregular streaks on upper and lower periphery. Aperture ovate; lip whitish and expanded; parietal callus transparent. Umbilicus rimate; columella whitish and expanded, overhanging umbilicus.

##### Remarks.

The shells of *Ganesella hariola*, *G. carinella* and *G. capitium* have a trochoid shape, but the shell of *G. capitium* is clearly more elevated than the shells of the two other species. The shell of *G. hariola* differs further from that of *G. capitium* and *G. carinella* in having a deep suture and a rounded last whorl with a brownish spiral band on the periphery. In contrast, the shells of *G. capitium* and *G. carinella* have a shallow suture and an angular to keeled last whorl without a brownish peripheral band.

#### Ganesella carinella

Taxon classificationAnimaliaStylommatophoraCamaenidae

(Möllendorff, 1902)

E4E7297D8C905B458E5741CCE3C20E43

**Figure 1.**
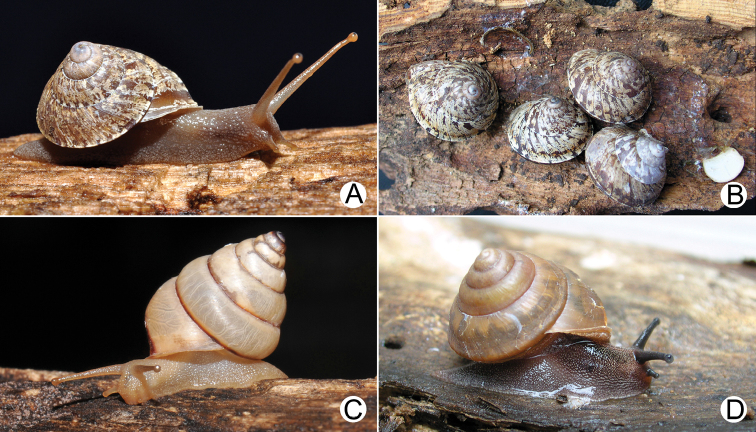
**A, B***Ganesella carinella* from Keng-khoy, Saraburi (shell width about 15 mm) **A** live snail and **B** snails aestivated under loose tree bark, and with white epiphrams attached on substrate **C***Ganesella rhombostoma* from Klong Had, Srakeo (shell height about 15 mm) **D***Globotrochus onestera* from Cuc Phuong, Vietnam (shell width about 15 mm).

**Figure 2.**
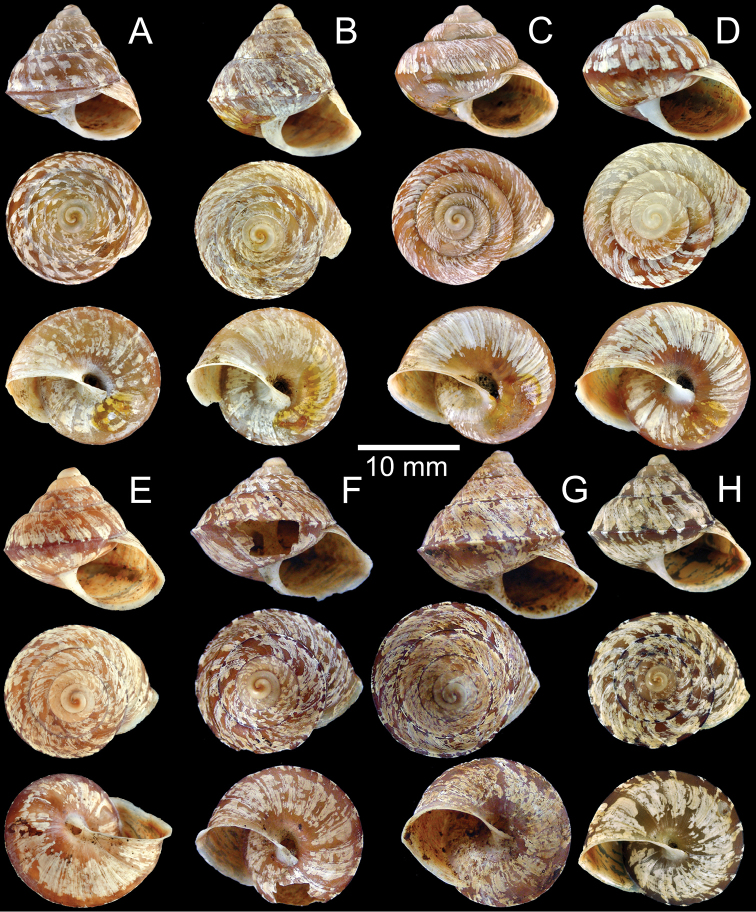
**A, B***Ganesella capitium***A** lectotype UMZC I.102385/1 and **B** paralectotype UMZC I.102385/2–3 **C–E***Ganesella hariola***C** lectotype UMZC I.104370/1 **D** paralectotype UMZC I.104370/2–4 and **E** shell from Popa Mountain, Myanmar CUMZ 5134 **F–H***Ganesella carinella***F** lectotype SMF 27534a **G** paralectotype SMF 27534b and **H** shell from Takhli, Nakhonsawan CUMZ 5133.

**Figure 3.**
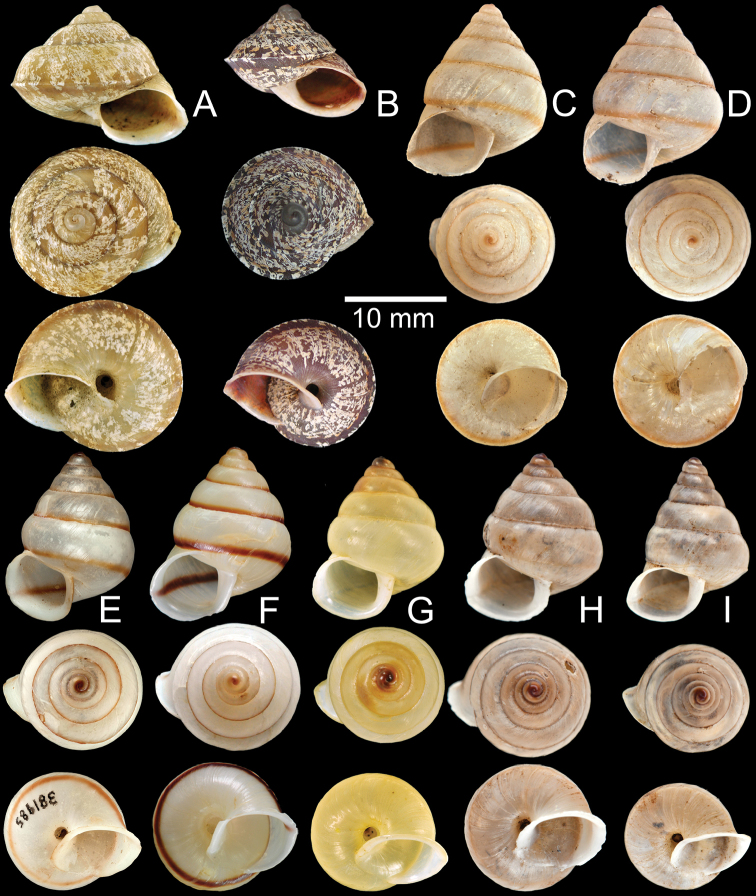
**A, B***Ganesella carinella***A** shell from Lopburi CUMZ 4282 and **B** shell from Tam Brichinda, Chiangmai CUMZ 4165 **C–I***Ganesella rhombostoma***C** lectotype NHMUK 20130215/1 **D** paralectotype NHMUK 20130215/2-3 **E** shell of “*harryleei* Thach, 2017” from Cambodia FMNH 381985 **F** shell from Klonghad, Srakeo CUMZ 5132 **G** shell from Chachoengsao CUMZ 5130 **H** shell from Sirisophon, Cambodia CUMZ 5131 and **I** shell from Srakaeo CUMZ 4286.

Figs 1A, B , 2F–H , 3A, B , 5A–D , 7A–C , 8

Eulota (Ganesella) hariola carinella Möllendorff, 1902: 158, 159. Zilch 1966: 202.
Ganesella capitium [non Benson 1848]: Pilsbry 1895: 170, 360, pl. 55, fig. 18. Blanford 1903: 278. Gude 1914: 196, 197. Zilch 1960: 610, fig. 2140. Zilch 1966: 202. Richardson 1985: 132, 133. Schileyko 2003: fig. 1958.

##### Type locality.

Siam, Muoklek and Kanburi [Thailand: Muaklek District, Saraburi Province and Kanchanaburi Province].

##### Material examined.

**Type specimens.** Lectotype SMF 27534a (Fig. 2F, height 14.8 mm, width 18.5 mm) and paralectotype SMF 27534b (1 shell; Fig. 2G, height 17.6 mm, width 18.2 mm) from Siam [Thailand].

##### Additional material.

**CAMBODIA**: Cambodia: NHMUK Cuming coll. ex Mouhot (2 shells). **THAILAND**: Siam: NHMUK 1902.9.17.30–31 (2 shells). Tam Barijinda, Chom Thong District, Chiangmai Province: CUMZ 4153, 4193, 4165 (Fig. 3B), 5123. Wat Tham Rakung, Sri Samrong District, Sukhothai Province: CUMZ 4937. Tam Lom-Tam Wang, Sri Samrong District, Sukhothai Province: CUMZ 4938. Tam Pha Thaphol, Nern Maprang District, Phitsanuloke Province: CUMZ 4195, 5127. Tam Wang Daeng, Nern Maprang District, Phitsanuloke Province: CUMZ 4932, 4939, 5113, 5126. Wat Chuek Charoentham, Ban Rai District, Uthaithani Province: CUMZ 4935. Wat Sri Uthumporn, Muang District, Nakhonsawan Province: CUMZ 4940. Tam Phet-Tam Thong, Takhi District, Nakhonsawan Province: CUMZ 4173, 4943, 5121, 5133 (Fig. 2H). Tam Poon Sawan, Srithep District, Phetchabun Province: CUMZ 4284. Tam Sombat Chomphol, Lomsak District, Phetchabun Province: CUMZ 4934, 5115, 5125. Khao Samokorn, Tha Wung District, Lopburi Province: CUMZ, 4218, 4279, 4282 (Fig. 3A), 4933. Wat Bandai Samsaen, Banmee District, Lopburi Province: CUMZ 4280. Tam Santisuk, Kok Samrong District, Lopburi Province: CUMZ 4931. Tam Tam-bon, Chaibadan District, Lopburi Province: CUMZ 5116. Muak Lek Waterfall, Muak Lek District, Saraburi Province: CUMZ 4186, 4172, 4941. Tam Dao Khaokaeo, Muak Lek District, Saraburi Province: CUMZ 4197. Tam Singha Ratde-cho, Kaeng Khoi District, Saraburi Province: CUMZ 4164, 4178, 5122. Tam Sriwilai, Chaloem Phrakiat District, Saraburi Province: CUMZ 4187, 4930. Wat Thep Pitak, Pakchong District, Nakhon Ratchasrima Province: CUMZ 4199. Wang Takrai Waterfall, Muang District, Nakhon Nayok Province: CUMZ 4942. Wat Khao Chakan, Khao Chakan District, Srakaeo Province: CUMZ 4159, 4182, 4213, 5114. Ta Praya District, Srakaeo Province: CUMZ 4283. Tam Phet Phothong, Klonghad District, Srakaeo Province: CUMZ 5120. Tam Kaeo Sawanbandan, Pong Namron District, Chanthaburi Province: CUMZ 4165.

##### Description.

**Shell.** Shell small, dextral, thin and depressed trochoid. Apex acute; embryonic shell smooth with brownish colour. Whorls 5-6, increasing regularly, slightly convex and slightly shouldered near suture; suture wide and shallow. Shell surface smooth or with fine growth lines. Last whorl large, angular with strong keel; beneath convex. Shell brownish and translucent, with wide, whitish-opaque, irregular spiral band on upper and lower side. Aperture ovate; lip slightly expanded and whitish; parietal callus transparent. Umbilicus rimate with small hole; columella whitish and expanded overhanging umbilicus.

**Genital organs.** Atrium (at) short (*N* = 10) and proximally with blackish tissues. Penis (p) cylindrical, about as long as the vagina; proximally blackish; distally slightly enlarged at penial verge. Epiphallus (e) and flagellum (fl) very short, approximately one-third of penis length. Vas deferens (vd) long and narrow, extends from free oviduct and terminates at distal epiphallus. Penial retractor muscle (pr) long and thin. Flagellum short, as long as or longer than epiphallus (Fig. 5A).

Penial verge (pv) small, conical, and with smooth surface. Penial wall ribbed, forming a series of swollen longitudinal pilasters (pp); middle wall with very thin pilasters. Pilasters swollen in the distal portion of penial chamber (Fig. 5B).

Vagina (v) long, cylindrical and proximally with blackish tissues. Gametolytic duct (gd) short; proximally wider; distally tapering to small tube and terminated with gametolytic sac (gc). Free oviduct (fo) short; oviduct (ov) enlarged with curled lobules. Albumen gland (ag) curved ligulate. Hermaphroditic duct (hd) convoluted and connected to hermaphrodic gland (hg) (Fig. 5A).

Internal wall of vagina with smooth, longitudinal, vaginal pilasters (vp); with slightly deep crenulated ridges throughout vaginal chamber (Fig. 5B).

**Radula.** Teeth arranged in nearly straight rows, each row containing 74 (37-(4-6)-1-(4-6)-36) teeth. Central tooth monocuspid with spatulate and truncate cusp. Lateral teeth larger than central tooth; teeth no. 1-3 monocuspid, no. 4-6 bicuspid, endocone spatulate and ectocone very small with pointed cusp (Fig. 7A, B). Marginal teeth start from teeth no. 6-7. Inner marginal teeth tricuspid, endocone and ectocone very small with pointed cusp and mesocone large and spatulate. Outermost teeth (near radula edge) tricuspid, endocone and ectocone with two or more pointed cusps, and mesocone large with curved cusp (Fig. 7B, C).

Jaw crescent, with anterior convex cutting margin. Vertical ribs prominent, and variable in number and size (Fig. 5D).

**Pallial system.** Typical sigmurethran; heart (au and ve) located left of kidney (on right in Fig. 5C). Pulmonary cavity approximately 5× longer than wide. Pulmonary vein (puv) and venation on lung roof (l) distinct and well developed. Kidney (k) long, slender and extending from posterior side to approximately the middle of pulmonary cavity. Ureter (ur) is a sigmoid, closed tube arising from apex of kidney, extending along right side of kidney, recurving near rectum (r). Rectal opening adjacent to anus (a) and pneumostome (pn) (Fig. 5C).

##### Distribution.

*Ganesella carinella* is widely distributed in Thailand: northern area in Chiangmai, Phitsanuloke; northeastern area in Loei, Phetchaboon, Nakhonratchasrima; central area in Saraburi, Lopburi; eastern area in Srakeow, Chanthaburi; western area in Kanchanaburi.

##### Remarks.

Hitherto, *Ganesella carinella* was regarded as a junior synonym of *G. capitium*. However, after Budha et al. (2012) described the genital apparatus of conchologically typical *G. capitium*, it became clear that *G. carinella* differs from the type species not only by having a more depressed shell with a strong peripheral keel but also by its longer penis and epiphallus, and its shorter vagina. In contrast, *G. capitium* has a more elevated trochoid shell, an angular last whorl, a shorter penis and epiphallus, and a longer vagina.

*Ganesella carinella* shows considerable variation in shell shape and colour. For example, specimens from Lopburi (Fig. 3A) tend to be paler and have a more descending aperture than specimens from Saraburi. Specimens from Chiangmai (Fig. 3B) tend to have more whitish spots on their shell than specimens from Lopburi and Saraburi. Yet, this conchological variation is not matched by consistent genital differences. Therefore, we conclude that the shell variation within this widely-distributed species only involves intraspecific polymorphism.

#### Ganesella rhombostoma

Taxon classificationAnimaliaStylommatophoraCamaenidae

(Pfeiffer, 1861)

3DA698469F775B358F4445E647431D68

Figs 1C , 3C–I , 5E–G , 7D–F , 8

Bulimus rhombostomus Pfeiffer, 1861: 194, 195. Pfeiffer 1868: 33.
Amphidromus rhombostomus : Pfeiffer and Clessin 1881: 214. Morlet 1889: 127. Morlet 1890: 121, 122, pl. 3, figs 6, 6a, b. Fischer and Dautzenberg 1904: 407.
Buliminus rhombostomus : Dautzenberg and Fischer 1906: 366, 367.
Buliminus rhombostomus var. pupoidea Dautzenberg and Fischer 1906: 367. Type locality: Hong-Chon, Cochinchine.
Giardia rhombostoma : Schileyko 2011: 46.
Pseudobuliminus harryleei Thach, 2017: 54, 55, figs 756–760. Type locality: Suburb of Battambang City, Battambang Province, Northwest Cambodia. New Synonym
Pseudobuliminus tuongvyae Thach, 2017: 56, figs 751–755. Type locality: Ha Tien, Kien Gaing Province, Southwest Vietnam. New Synonym
Pseudobuliminus huberi Thach, 2017: 55, figs 759–760. Type locality: 20 km of Kampong Trach District, Kampot Province, Northwest Cambodia. New Synonym

##### Type locality.

Camboja [Cambodia].

##### Material examined.

**Type specimens.** Three syntypes of *Bulimus rhombostomus* Pfeiffer, 1861 in H. Cuming collection, the shell that best matches with the original description is designated here as the lectotype NHMUK 20130215/1 (Fig. 3C, height 16.7 mm, width 11.5 mm) to stabilize the name; the other two shells from the same lot become the paralectotypes NHMUK 20130215/2-3 (2 shells; Fig. 3D, height 18.4 mm, width 13.2 mm).

##### Additional material.

**VIETNAM**: Ha Tien, Kien Gaing Province, Southwest Vietnam: holotype of *Pseudobuliminus tuongvyae* Thach, 2017 MNHN-IM-2000-33203. **CAMBODIA**: Suburb of Battambang City, Battambang Province, Northwest Cambodia: holotype of *Pseudobuliminus harryleei* Thach, 2017 FMNH 381985 (Fig. 3E). Wat Thammaban Khiri, Sirisophon Town, Banteay Meanchey Province (13°37'58.1"N, 102°56'38.0"E): CUMZ 5131 (Fig. 3H). **THAILAND**: Tam Leoum, Klonghad District, Srakaeo Province: CUMZ 4286 (Fig. 3I). Tam Pha Pheung, Klonghad District, Srakaeo Province: CUMZ 5124. Tam Srithong, Klonghad District, Srakaeo Province: CUMZ 4070, 5118, 5132 (Fig. 3F). Tam Phet Phothong, Klonghad District, Srakaeo Province: CUMZ 4600, 5119. Khoa Phlapphueng Thong, Wang Somboon District, Srakaeo Province: CUMZ 4069. Wat Khao Maka, Muang District, Srakaeo Province: CUMZ 4071, 4598, 4599, 5130 (Fig. 3G). Tam Kaeo Sawanbandan, Pong Namron District, Chanthaburi Province: CUMZ 4285.Tam Rad, Tha Takiep District, Chachoengsao Province: CUMZ 5117.

##### Description.

**Shell.** Shell small, sinistral, thin and trochoid. Apex acute with blackish colour; embryonic shell smooth. Whorls 5-6, increasing regularly, convex; suture wide and shallow. Shell surface smooth or with fine growth lines. Last whorl large, well rounded, keeled near aperture; with or without brownish spiral band. Shell monochrome white, yellow to light brownish and translucent. Aperture semi-ovate, open subventrally; lip expanded and whitish; parietal callus transparent. Umbilicus rimate; columella wide and whitish.

**Genital organs.** Atrium (at) short (*N* = 10). Penis (p) cylindrical, long and may be as long as the vagina. Epiphallus (e) and flagellum (fl) each about half as long as the penis. Vas deferens (vd) long and narrow, extending from free oviduct and connected to distal part of epiphallus. Penial retractor muscle (pr) long and slightly thickened (Fig. 5E).

Penial verge (pv) small, conical, and with smooth surface. Penial wall ribbed, forming a series of irregular, smooth longitudinal pilasters (pp) that encircle penial verge (Fig. 5F).

Vagina (v) large, cylindrical about as long as penis. Gametolytic duct (gd) short, cylindrical, gradually tapering towards gametolytic sac (gs). Free oviduct (fo) short, about half as long as vagina; oviduct (ov) enlarged with curled lobules. Albumen gland (ag) curved ligulate. Hermaphroditic duct (hd) convoluted and connected to hermaphrodic gland (hg) (Fig. 5E).

Vaginal wall with several smooth, longitudinal pilasters; vaginal wall itself with strong longitudinal ridges through the vaginal chamber (Fig. 5F).

**Radula.** Teeth arranged in nearly straight rows, each row containing 58 (29-(6-9)-1-(7-9)-28) teeth. Central tooth triangular, symmetric monocuspid with dull cusp. Lateral teeth asymmetric, teeth no. 1-6 monocuspid and no. 7-10 bicuspid with very small ectocone (Fig. 7D, E). Marginal teeth start from teeth no. 9-10, tricuspid, endocone very small to absent, mesocone large triangular, and ectocone very small with pointed cusp. Outermost marginal teeth (near radula edge) tricuspid, endocone small, mesocone large with curved cusp, and ectocone with one, two or more pointed cusps (Fig. 7E, F).

Jaw crescent, with anteriorly convex cutting margin. Vertical ribs thin, and variable in number and size (Fig. 5G).

##### Distribution.

This species was formerly known from its type locality in Cambodia (Morlet 1889, 1890) and some inaccurate localities recorded from Vietnam (Schileyko 2011). In Thailand, it is known from several localities in Chachoengsao, Srakeo and Chanthaburi Provinces.

##### Remarks.

*Ganesella rhombostoma* has long been overlooked and its taxonomic status has been unclear. The species has been erroneously assigned to *Amphidromus* Albers, 1850 and *Giardia* Ancey, 1907 (see Dautzenberg and Fischer 1906, Schileyko 2011). Yet, its trochoid shell and sub-ventrally opening aperture show that it belongs to neither of these genera, since *Giardia* (type species *Bulimus siamensis* Redfield, 1853) and *Amphidromus* (type species *Helix perversus* Linnaeus, 1758) have an elongate, ovate shell, a non-deflected last whorl, an ovate and laterally opening aperture, and a narrowly opened umbilicus (see also Schileyko (2003) and Sutcharit and Panha (2006) for further comparisons). Moreover, the anatomical evidence presented here suggests that this species rather belongs to *Ganesella*. However, the relationships between *Ganesella* and *Giardia* needs further investigation by molecular analysis.

Specimens from isolated limestone outcrops at Khao Maka, Chacheongsao are smaller and have a more ovate shell without a brownish spiral band on of the last whorl (Fig. 3G). However, anatomically they are indistinguishable from typical *G. rhombostoma* and, therefore, they are considered conspecific.

#### Ganesella halabalah

Taxon classificationAnimaliaStylommatophoraCamaenidae

Sutcharit & Panha sp. nov.

5DCCA8AC5A4C549BB284EE5CF0D4B4CC

http://zoobank.org/65AC036B-D3B7-4AE4-A363-5496D1F42146

Figures 4A–C , 8

##### Type material.

**Figure 4.**
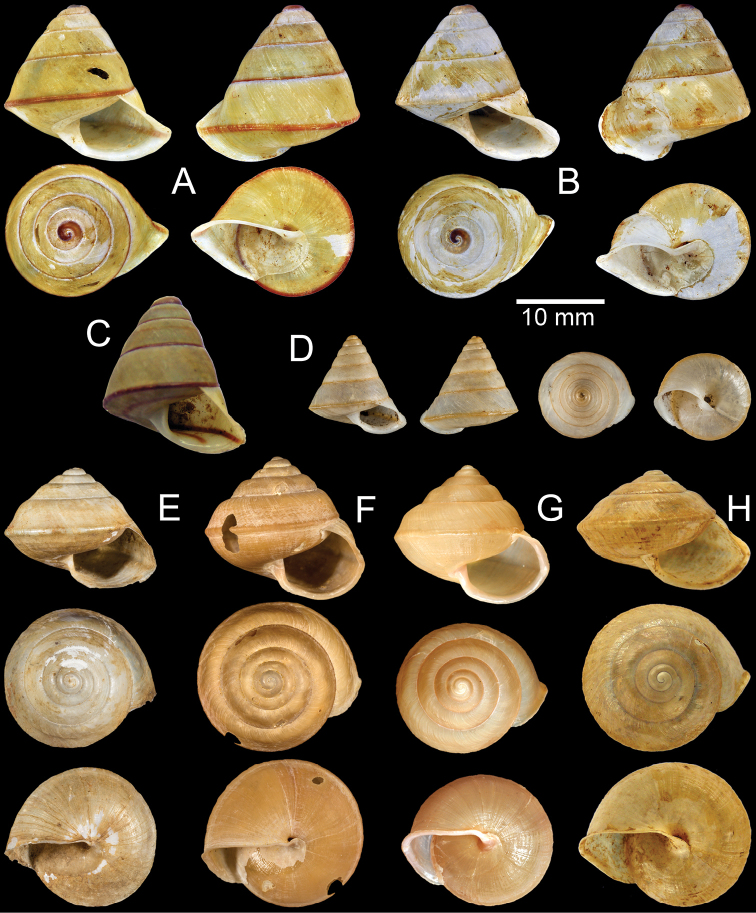
**A–C***Ganesella halabalah* sp. nov. **A** holotype CUMZ 2608 **B** paratype CUMZ 2599 from the type locality and **C** paratype ZMB 53120 from Kelantan, Malaysia **D***Ganesella perakensis*, syntype MNHN-IM-2000-1964. **E–G***Globotrochus onestera***E** lectotype MNHN-IM-2000-32456 **F** holotype of “*simonei* Thach & Huber, 2017” MNHN-IM-2000-33206 and **G** specimen from Vietnam CUMZ 5218 **H***Globotrochus mellea*, holotype RBINS/MT/ 525051.

Holotype CUMZ 2608 (Fig. 4A, height 22.6 mm, width 23.3 mm, 5¾ whorls), paratypes CUMZ 2599 (3 shells; Fig. 4B, height 22.0 mm, width 22.1 mm) from the type locality. Paratype ZMB 53120 (1 shell; Fig. 4C) ex. Waterstradt coll. from Gunung Tahan, Kelantan, Malaysia.

##### Type locality.

Sirindhorn Waterfall, Hala-Bala Wildlife Sanctuary, Waeng District, Narathivat Province, Thailand.

##### Diagnosis.

The dextral, large, trocoid shell with pale green to yellow colour, obtuse apex and apertural lip with prominent beak-like deflection.

##### Description.

**Shell.** Shell medium-sized (height 23.6 mm, width 25.1 mm), thin, dextral and trochoid. Apex obtuse; embryonic shell smooth and black. Whorls 5-6, increasing regularly, smooth; suture wide and shallow; shell surface with thin growth lines. Last whorl large, with well-developed peripheral keel and blunt at lower periphery. Shell colour pale green or yellow to monochrome creamy; earlier whorls paler; with or without brown spiral band on peripheral keel and lower periphery. Periostracum thin corneous, brownish and translucent. Aperture relatively large, semi-ovate; parietal callus transparent; columella wide and whitish. Apertural lip expanded, whitish, and angled with prominent beak-like deflection at peripheral keel. Umbilicus rimate and partially obscured by lower apertural lip.

##### Etymology.

The specific name is derived from the type locality Hala-Bala Wildlife Sanctuary, Narathivat, Thailand.

##### Distribution.

This new species is currently known from the type locality (in Narathivat, Thailand) and Gunung Tahan, Kelantan, Malaysia, which is about 150 km south of the type locality. The latter shell (Fig. 4C) was collected in 1901 in a tropical rain forest. This shell is in all aspects identical to the unique name-bearing type.

##### Remarks.

Even though *Ganesella halabalah* sp. nov. is described from empty shells, its unique features mean that it cannot be confused with any other camaenid species from the area. Yet, with its trochoid shell and its prominent, beak-like apertural rostrum, *G. halabalah* sp. nov. does resemble a Papuininae phenotype. However, the geographic distribution of the Papuininae is largely restricted to New Guinea, Australia and Melanesia (Schileyko 2003), though excluding the Greater Sunda Islands and Indochina. Given that the Malay Peninsula is a remote area for land snail dispersal between Australasia and Indochina (Hausdorf 2000), further anatomical and molecular evidence is needed to assess an eventual relationship with Papuininae.

This new species clearly differs from all *Ganesella* and other land snail species known in Indochina. The most similar species is *Ganesella perakensis* (Crosse, 1879) from Malaysia, which has a much smaller (average shell height < 10 mm), thin shell, and a simple apertural lip (Fig. 4D). *Ganesella halabalah* sp. nov. has a larger shell (average shell height > 20 mm), an obtuse apex and an aperture lip with a typical, prominent beak-like deflection.

The new species also differs from all *Kenyirus* Clements & Tan, 2012 species from Malaysia by having a conical spire, yellowish shell and narrower umbilicus. While *K. sodhii* Clements & Tan, 2012 has a depressed spire, long spout-like apertural rostrum on the peripheral keel, and 3-4 brownish spiral bands on the last whorl. In comparison *K. sheema* Foon et al., 2015 has subglobose shell, an angular last whorl, and with two brownish spiral bands below the periphery; while *K. balingensis* Tan et al., 2017 has a smaller and brownish shell.

**Figure 5.**
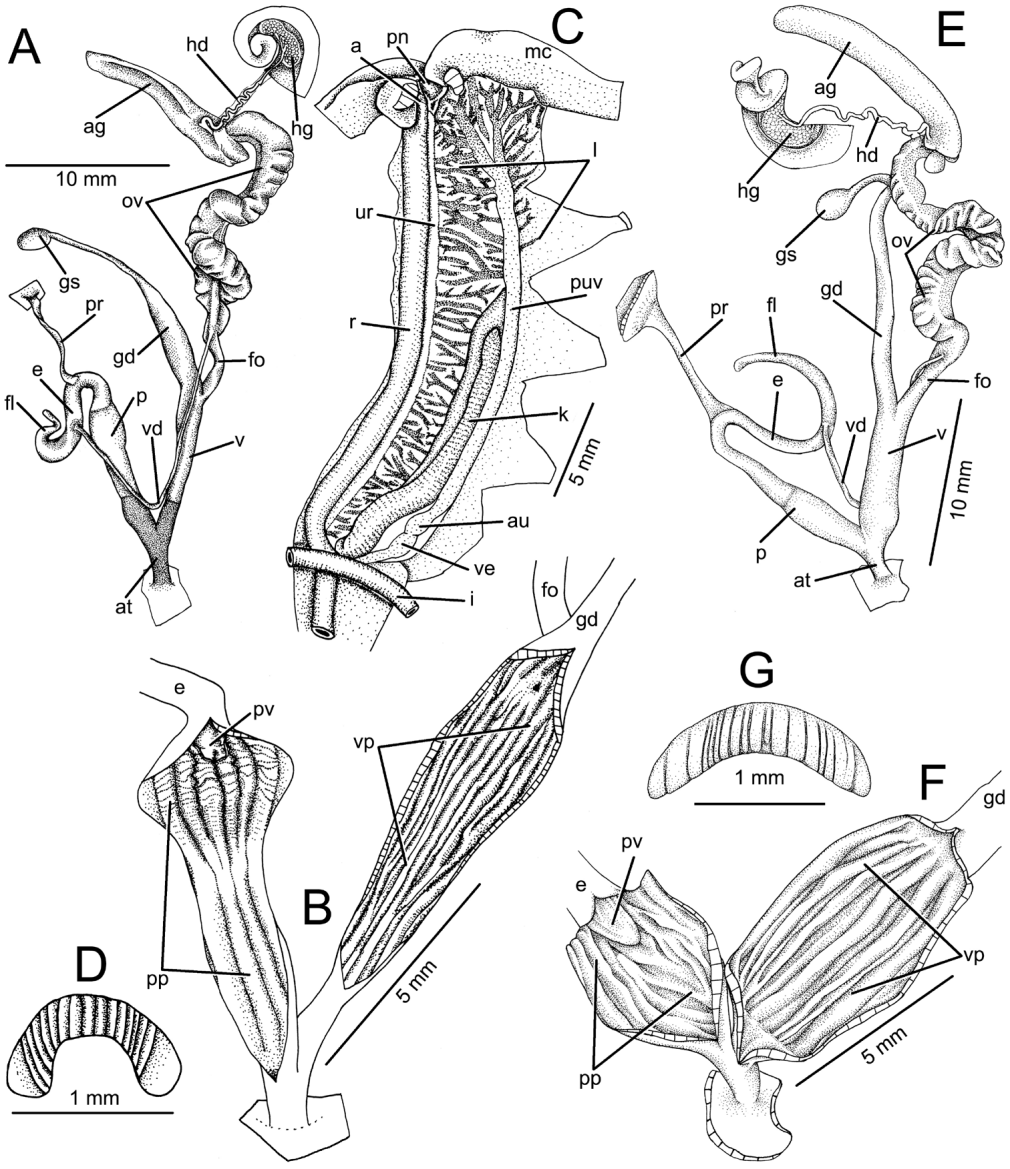
**A–D***Ganesella carinella* from Saraburi **A** general view of genital system **B** internal structure of penis and vagina **C** pallial system and pulmonary cavity and **D** jaw **E–G***Ganesella rhombostoma* from Srakaeo **E** general view of genital system **F** internal structure of penis and vagina and **G** jaw.

#### Genus Globotrochus

Taxon classificationAnimaliaStylommatophoraCamaenidae

Haas, 1935

A693D35D5A785082898FBDE1A3D2BC12

Ganesella (Globotrochus) Haas, 1935: 47. Zilch 1960: 611. Zilch 1966: 210.
Globotrochus : Schileyko 2003: 1519. Schileyko 2011: 46.

##### Type species.

*Helix onestera* Mabille, 1887, by monotypy.

##### Description.

Shell trochoid, thin, translucent, 4-6 slightly convex whorls. Last whorl angulated or carinated, slightly descending in front. Colour light yellowish-brown. Embryonic shell smooth. Post apical whorls with irregular, thin, radial ridges and spiral line (smooth below peripheral angle or keel). Aperture ovate, moderately oblique, with slightly expanded and reflexed margins. Umbilicus closed. Shell height ranged from 16 to 17 mm and shell width ranged from 21 to 22 mm.

Genitalia typical of camaenids, but with thin (small size and fully functional) male genital organs (penis and epiphallus).

Radular teeth triangular and tricuspid.

##### Remarks.

The weak development of male genitalia in *Globotrochus* is unusual among the Indochinese taxa, but is common in papuinid genera, such as *Papustyla* Pilsbry, 1893, *Letitia* Iredale, 1941, *Papunella* Clench & Turner, 1959 and *Wahgia* Clench & Turner, 1959 (Schileyko 2003). However, Papuininae are geographically confined to New Guinea, Australia and Melanesia (Schileyko 2003), and have never been recorded in Indochina. Therefore, it seems as if ‘weak male genital parts’ is an autapomorphy of *Globotrochus*.

*Globotrochus* differs from all other camaenid genera in Indochina (i.e., *Chloritis*, *Satsuma*, *Ganesella* and *Neocepolis*) by its weakly developed male genital organs. In contrast, the genera *Chloritis*, *Ganesella* and *Neocepolis* have typical camaenid genitalia, with well-developed male genital organs, including a relatively short to long penis, an epiphallus and penial verge, and a short to long flagellum. Furthermore, *Ganesella* has no penial appendix. *Satsuma* has a fully-developed male genital organ, with a short to long penial appendage, but without a penial verge (Solem 1993, Schileyko 2003, 2004, Sutcharit et al. 2007, Sutcharit and Panha 2010).

Currently, only two nominal species are assigned to *Globotrochus*. Based on the literature, museum specimens and recent field surveys, it seems as if *Globotrochus* is restricted to the north of Vietnam (Vermeulen and Maassen 2003, Schileyko 2003, 2011). However, an ambiguous locality record from Elephant Mountain, Laos (sensu Schileyko 2011: 46) needs verification.

#### Globotrochus onestera

Taxon classificationAnimaliaStylommatophoraCamaenidae

(Mabille, 1887)

F42E007E12175C8FBADD1AB02D8613DB

**Figure 6.**
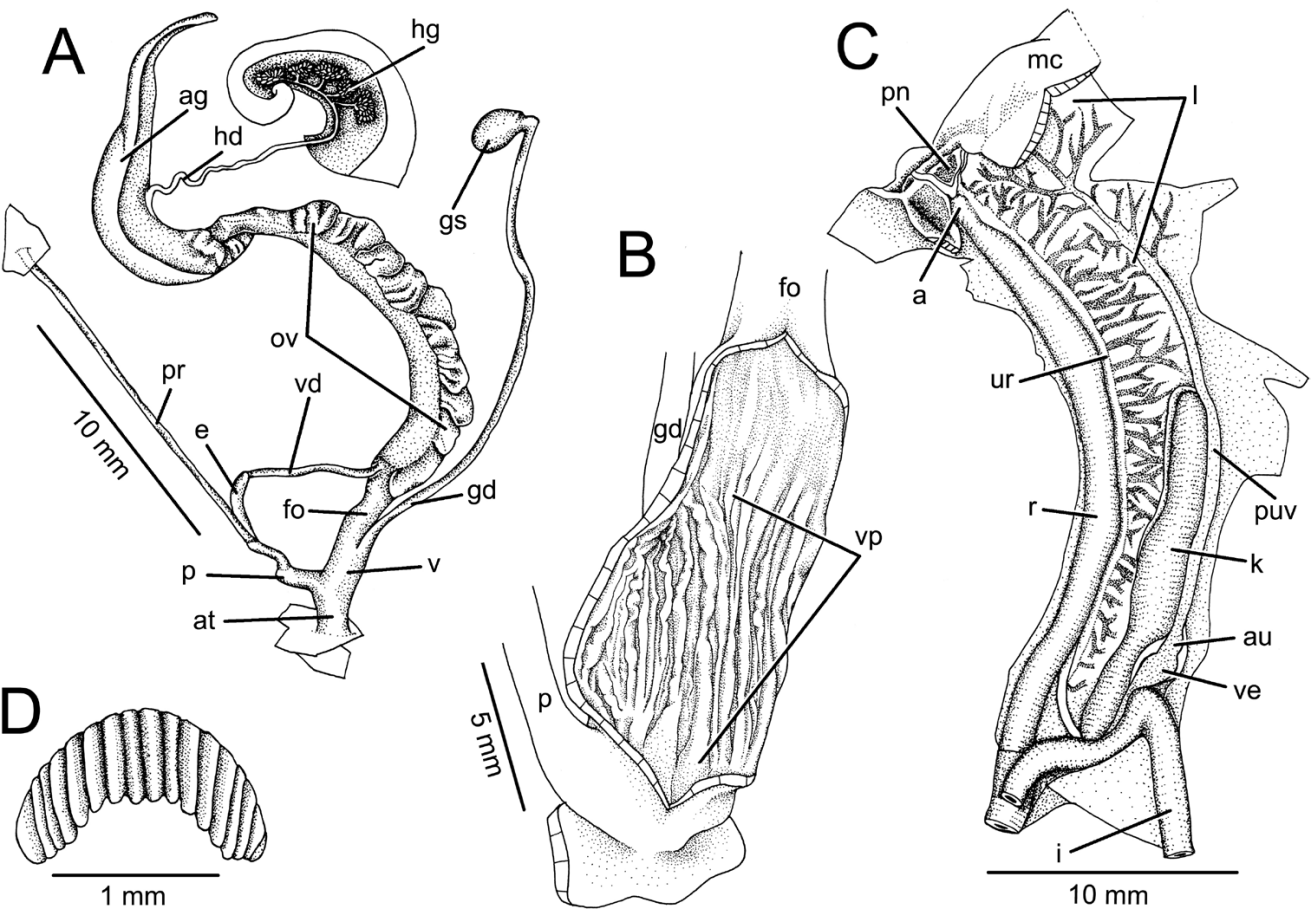
Anatomy of *Globotrochus onestera* from Cuc Phuong, Vietnam. **A** general view of genital system **B** internal structure of vaginal chamber **C** pallial system and pulmonary cavity and **D** jaw.

**Figure 7.**
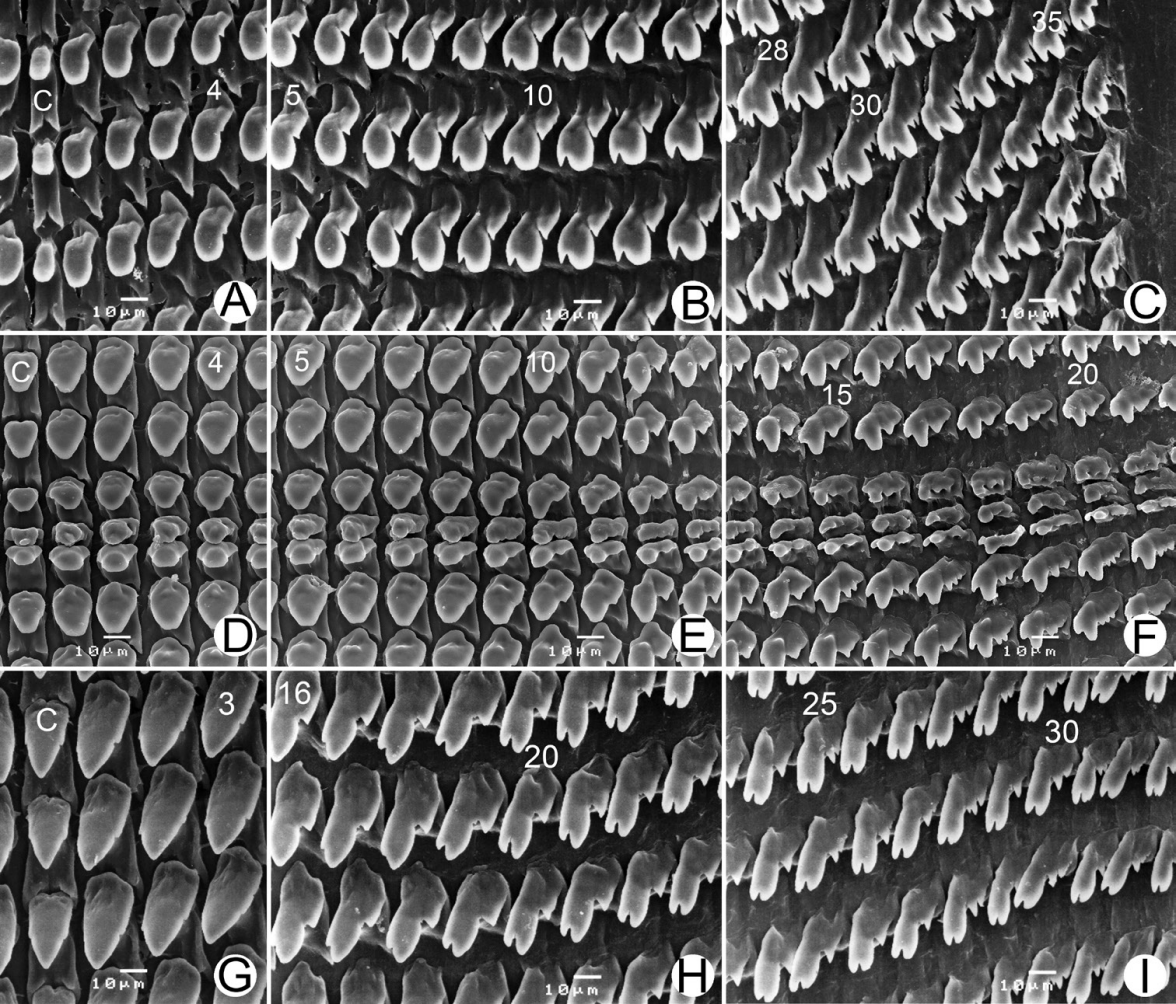
Radula. **A–C***Ganesella carinella***D–F***Ganesella rhombostoma* and **G–I***Globotrochus onestera***A, D, G** central tooth with the first to the second lateral teeth **B, E, H** lateral teeth with the tricuspid marginal teeth transition **C, F, I** outermost marginal teeth. Numbers indicate order of lateral and marginal teeth. Central tooth indicated by ‘C’.

**Figure 8.**
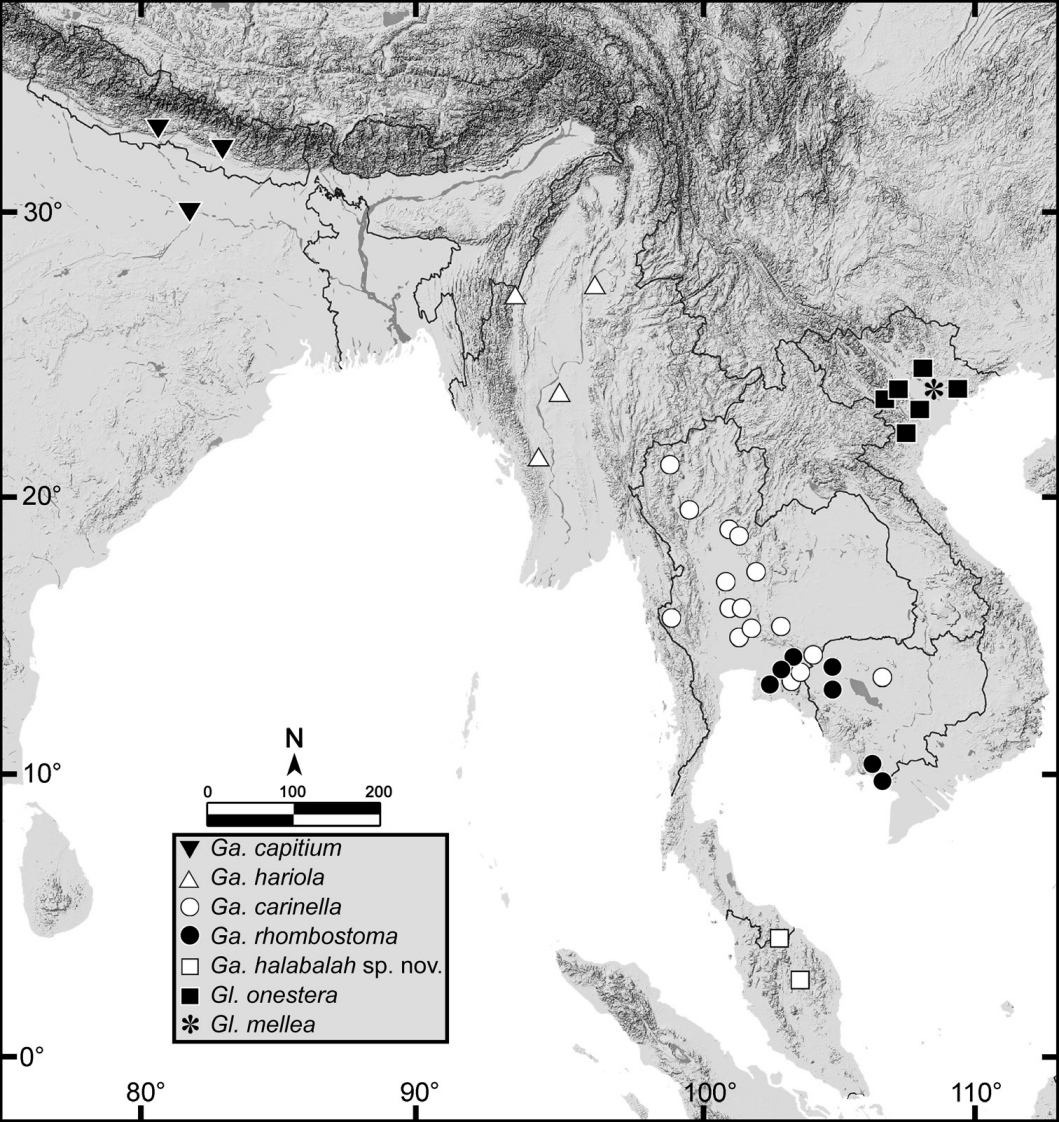
Geographic distribution of *Ganesella* spp. and *Globotrochus* spp.

Figs 1D , 4E–G , 6 , 7G–I , 8

Helix onestera Mabille, 1887a: 3. Mabille 1887b: 89, 90, pl. 2, figs 4, 5. Dautzenberg and Fischer 1908: 184, 185. Haas 1935: 46, 47. Fischer and Dautzenberg 1904: 404.
Ganesella (Globotrochus) onestera : Zilch 1960: 611, fig. 2143. Zilch 1966: 210.
Ganesella onestera : Richardson 1985: 140.
Globotrochus onestera : Schileyko 2003: 1519, fig. 1959. Schileyko 2011: 46. Inkhavilay et al. 2019: 152, fig. 60c.
Diastole simonei Thach & Huber in Thach 2017: 34, 35, figs 418–420. Type locality: Nho Quan District, Ninh Binh Province, North Vietnam. New Synonym.

##### Type locality.

Tonkin [north Vietnam].

##### Material examined.

**Type material**. This species was described on the basis of shells from the Balansa coll. Mabille (1887b: 89, 90, pl. 2, figs 4, 5) re-published the description of the species with an illustration of a shell. The MNHN-Malacologie collection contains two lots with syntypes. The first lot is from the Balansa coll. and consists of two shells with an original label giving the taxon name, collection locality and marked with the word “M. Balansa 1887. Type”. One of the two shells is not damaged and its size closely matches the measurements in the original description and the illustration of Mabille (1887b: pl. 2, figs 4, 5). This shell is, therefore, here designated as the lectotype MNHN-IM-2000-32456/1 (Fig. 4E, height 15.1 mm, width 20.5 mm, 5 whorls). The second shell is broken up into three pieces and becomes the paralectoype MNHN-IM-2000-32456/2 (1 shell). The second lot MNHN-IM-2000-2073 consists of a shell with the marking “Type” on its label. Yet, this lot does not belong to the Balansa collection and was subsequently labeled as “*H. onestera* J. Mab. var.”. Therefore, we exclude this lot from the type series of this nominal species (ICZN 1999: Art. 72.4.1).

##### Other material.

**VIETNAM**: Nho Quan, District, Ninh Binh Province, North Vietnam: holotype of *Diastole simonei* Thach & Huber, 2017 MNHN-IM-2000-33206 (1 shell; Fig. 4F). Buc-Kan, Tonkin: NHMUK Kennard coll. (1 shell), MNHN (1 juvenile shell). Haiphong, Tonkin: NHMUK 1893.12.8.21–22 (2 shells), NHMUK Salisbury ex Beddome coll. (2 shells), SMF 27512 (3 shells), SMF 297452 (2 shells), ZMB 47931 (1 shells), NHMW 23331 (1 shell). Nin-Cho, Nga Ba Tha, Tonkin: SMF 297450 (3 shells), NHMW 11734 (2 shells), NHMW 50818 (2 shells), NHMW Rusnov coll. (2 shells). Da-Bac, Tonkin: SMF 297451 (3 shells). Cuc Phuong National Park, Nho Quan District, Ninh Binh Province (20°14'59.0"N, 105°42'52.3"E): CUMZ 5128 (Fig. 4G), 5129.

##### Description.

**Shell.** Shell medium-sized, dextral, thin and trochoid. Apex acute; embryonic shell smooth with brownish colour. Whorls 5-6, increasing regularly, slightly convex and slightly shouldered near suture; suture wide and shallow. Shell surface smooth or with fine growth lines. Last whorl large, angular with strong keel, beneath slightly convex. Shell monochrome, light brownish and translucent. Aperture semi-ovate; lip slightly expanded and brownish; parietal callus thin. Umbilicus closed; columella small and whitish.

**Genitalia.** Male genital organ (*N* = 3) thin (small size and fully function). Atrium (at) short, about as long as penis. Penis (p) short, proximally cylindrical, distally like a short, but wider tube. Penial sheath and penial verge absent. Epiphallus (e) small, as long as penis; flagellum absent. Vas deferens (vd), short, narrow, extending from free oviduct to tip of epiphallus. Penial retractor muscle (pr) relatively thin and long (Fig. 6A).

Vagina (v), cylindrical, short, size and shape similar to atrium. Gametolytic duct (gd) long and narrow; distally terminating at gametolytic sac (gs). Free oviduct (fo) relatively long; oviduct widened by curled lobules. Albumen gland (ag) curved lingulate. Hermaphroditic duct (hd) convoluted and located between hermaphrodic gland (hg) (Fig. 6A).

Internal walls of vagina with several smooth surfaces of longitudinal pilasters (vp). Vaginal wall itself with strong longitudinal ridges through the vaginal chamber (Fig. 6B).

**Radula.** Teeth arranged in anteriorly pointed, nearly straight rows, each row containing about 79 (39-1-39) teeth. Central tooth symmetric tricuspid, mesocone large with pointed cusp, ectocone very small and located in the middle of tooth. Lateral and marginal teeth undivided. Inner teeth (no. 1-14) asymmetric tricuspid, endocone and ectocone small, and mesocone large with pointed cusp (Fig. 7G, H). Outermost teeth tricuspid, endocone small and located close to apex of teeth; mesocone relatively large with curved cusp, and ectocone located at base and cusps sometimes split into two pointed cusps (Fig. 7H, I).

Jaw crescent, with anteriorly convex cutting margin. Vertical ribs prominent, variable in number and size (Fig. 6D).

**Pallial system.** Typical sigmurethran; heart (auricle and ventricle) located left of kidney (on right in Fig. 6C). Pulmonary cavity approximately 5× longer than wide. Pulmonary vein and venation on lung roof distinct and well developed. Kidney elongated, slender and extending from posterior side of cavity to approximately half of pulmonary cavity. Ureter sigmoidal, closed tube arising from apex of kidney, extending along right side of kidney, recurving adjacent to rectum. Rectal opening adjacent to anus and mantle collar (Fig. 6C).

##### Distribution.

This species is known from the type locality and recent records from Cuc Phuong National Park, Ninh Binh, and Nui Con Vui near Hai Phong, in northern Vietnam (Schileyko 2011).

##### Remark.

Live specimens of this species were collected for the first time in 2006 at Cuc Phuong, Vietnam. This is a very humid area with tropical forest patches and limestone karst. The snails were collected on small shrubs, suggesting that it may be an arboreal species.

Recently, Thach (2017) described a new species from Vietnam under the southern Pacific Islands endemic genus *Diastole* Gude, 1913 (see Schileyko 2002). The species *Diastole simonei* Thach & Huber, 2017 was described from the same geographical area as *Globotrochus onestera*, the original description of which did not mention this species. The type specimens of *Globotrochus onestera* and *Diastole simonei* are identical in all characters. Hence, we treat *Diastole simonei* Thach & Huber, 2017 as a junior subjective synonym of *Globotrochus onestera*.

#### Globotrochus mellea

Taxon classificationAnimaliaStylommatophoraCamaenidae

(Bavay & Dautzenberg, 1915)

170486F9986A5436B9B050EC0499FCEF

Figs 4H , 8

Helix (Ganesella?) mellea Bavay & Dautzenberg, 1915: 147, 148, pl. 5, figs 1–3.
Ganesella mellea : Richardson 1985: 139.
Globotrochus mellea : Schileyko 2011: 46.

##### Type locality.

Nui-Ba-Dinh, Phu-Ha, and Phu-Ly [Vietnam].

##### Material examined.

**Type material.** Only a single shell was available, viz. the syntype RBINS/MT/ 525051 ex. Dautzenberg collection (1 shell; Fig. 4H, height 18.4 mm, width 26.3 mm).

##### Description.

**Shell.** Shell medium-sized, dextral, thin and depressed trochoid. Apex acute; embryonic shell smooth. Whorls 5-6, increasing regularly, slightly convex and slightly shouldered near suture; suture wide and shallow. Shell surface with fine growth lines. Last whorl large, angular with strong peripheral keel; slightly convex beneath. Shell monochrome, light brownish and translucent. Aperture semi-ovate; lip slightly expanded and brownish; parietal callus thin. Umbilicus closed; columella small and whitish.

##### Remark.

*Globotrochus mellea* is similar to *Globotrochus onestera* in almost all shell characters, except for its larger last whorl, strong angular peripheral keel and more depressed trochoid shell. Therefore, we provisionally retain *Globotrochus mellea* as a distinct species.

## Supplementary Material

XML Treatment for Ganesella

XML Treatment for Ganesella capitium

XML Treatment for Ganesella hariola

XML Treatment for Ganesella carinella

XML Treatment for Ganesella rhombostoma

XML Treatment for Ganesella halabalah

XML Treatment for Globotrochus

XML Treatment for Globotrochus onestera

XML Treatment for Globotrochus mellea
